# Ostéodystrophie héréditaire d'Albright: à propos d'une observation

**DOI:** 10.11604/pamj.2019.34.190.13398

**Published:** 2019-12-10

**Authors:** Laila Tami, Chaima Rherib, Kanza Chefchaouni, Houria Knouni, Amina Barkat

**Affiliations:** 1 Service de Néonatologie, Centre National de Référence en Néonatologie et en Nutrition, Hôpital d'Enfants, Rabat, CHU Ibn-Sina, Maroc; 2 Équipe de Recherche en Santé et Nutrition du Couple Mère-Enfant, Faculté de Médecine et de Pharmacie de Rabat, Université Mohammed V, Rabat, Maroc

**Keywords:** Ostéodystrophie héréditaire d´Albright, hypocalcémie, bilan phosphocalcique, calcifications, Albright's hereditary osteodystrophy, hypocalcaemia, phosphocalcic balance, calcifications

## Abstract

L'ostéodystrophie héréditaire d'Albright est une pathologie rare, associée à des troubles du bilan phosphocalcique liés à une résistance périphérique à la parathormone. Il s'agit d'une affection héréditaire, transmise sur le mode autosomique dominant et qui résulte d'une anomalie du gène GNAS1. Elle associe un morphotype particulier, des calcifications sous cutanées et une résistance osseuse et rénale à la parathormone. Nous rapportons un nouveau cas d'ostéodystrophie héréditaire d'Albright chez un nourrisson de 9 mois, suivi pour hypocalcémie profonde depuis j10 de vie. A travers ce cas, nous rappelons les différents aspects de cette affection sur les plans clinique, biologique, génétique ainsi que thérapeutique.

## Abstract (en)

Albright's hereditary osteodystrophy is a rare disease, characterized by phosphocalcic balance abnormalities related to peripheral resistance to parathyroid hormone. It is an hereditary affection with autosomal dominant inheritance pattern caused by mutation in GNAS gene1. It combines specific morphotype, subcutaneous calcifications, bone and renal resistance to parathyroid hormone. We report a new case of Albright's hereditary osteodystrophy in a 9-month old infant followed up for significant hypocalcaemia which occurred at 10 days of life. The purpose of this study was to remind clinicians of the clinical, biological, genetic and therapeutic features of this disease.

## Introduction

La pseudohypoparathyroïdie ou Ostéodystrophie Héréditaire d'Albright est le premier exemple de résistance hormonale observé en pathologie humaine. Albright, en 1942 [1], en fit la première description. Cette maladie génétique rare, associe un morphotype particulier, des calcifications sous-cutanées et une résistance osseuse et rénale à la parathormone. D'autres résistances hormonales peuvent également être présentes. Elle est liée à une mutation du gène GNASI, localisé en 20q13. 2q13.3 [2]. Une même mutation est responsable de plusieurs phénotypes en fonction de l'allèle parental hérité [3]: pseudo-hypoparathyroïdie de type 1a, 1b, 1c, pseudo-pseudohypoparathyroïdie (type 2), mais également d'une maladie voisine, l'hétéroplasie osseuse progressive (HOP). Nous présentons un cas de pseudohypoparathyroidie de type 1a. Un consentement parental est pris pour le rapport de ce cas.

## Patient et observation

**Figure 1:**
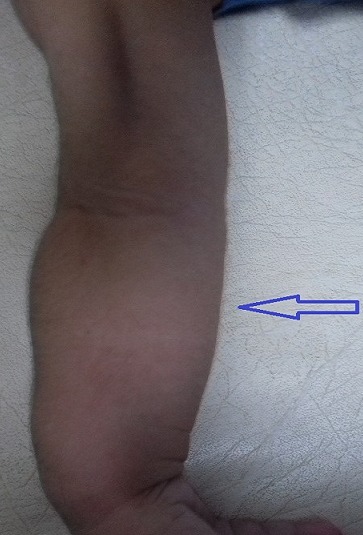
Avant-bras incurvés (flèche)

**Figure 2:**
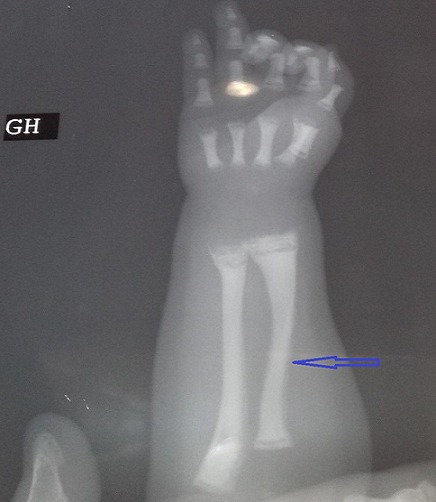
Incurvation des diaphyses radiales (flèche)

**Figure 3:**
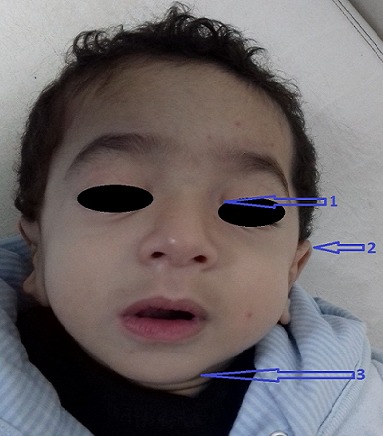
Faciès dysmorphique: A) épicanthus; B) oreilles bas implantés, C) retrognatisme

**Figure 4:**
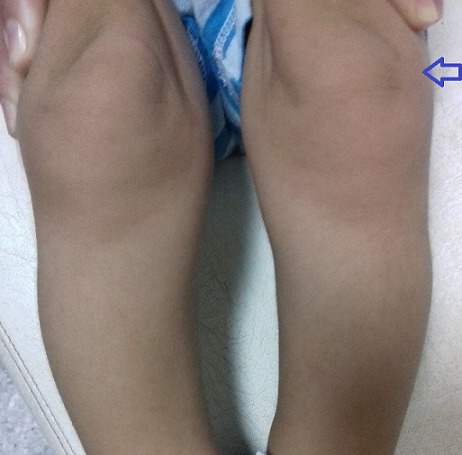
Genu varum (flèche)

M.A.Y. nourrisson de 9 mois, de sexe masculin, issu d'une grossesse suivie, menée à terme, accouchement par voie haute pour souffrance fœtale aigue sur double circulaire du cordon avec un apgar à 10/10/10. De parents consanguins 1^er^ degré, mère âgée de 40 ans, G4P3 (G3 avortement spontané, 3 enfants vivants), suivie pour thyroïdite d'hachimoto sous Levothyroxine. Le nourrisson est suivi depuis j10 de vie pour hypocalcémie sévère diagnostiquée suite à un état de mal convulsif. L'examen clinique a objectivé une hypotonie axiale, un retard des acquisitions psychomotrices, un syndrome polymalformatif fait d'un faciès dysmorphique (épicanthus, macroglossie, retrognatisme et oreilles bas implantées) (Figure 1), de genu varum (Figure 2) et d'avants-bras incurvés (Figure 3) avec absence de poursuite oculaire et rétrécissement coanal. Le bilan phosphocalcique est perturbé objectivant une hypocalcémie profonde réfractaire à la supplémentation calcique, une calciurie basse, une hyperphosphorémie avec des taux de parathormone élevés (Tableau 1). Le bilan malformatif a mis en évidence une altération bilatérale de la conduction rétino-cortinale (Amblyopie) et une incurvation des diaphyses radiales et fémorales avec trabéculations à larges mailles au niveau des extrémités des membres (Figure 4). L'échographie abdomino-rénale, l'échographie trans-thoracique, l'imagerie par résonnance magnétique et l'électroencéphalogramme sont revenus normaux. Devant ce tableau associant le syndrome poly malformatif aux troubles phosphocalciques, une pseudohypoparathyroidie et notamment type 1a avec une ostéodystrophie héréditaire d'Albright a été évoquée en premier. Le nourrisson est mis sous supplémentation de calcium avec suivi clinique et biologique. Une étude génétique est en cours.

**Tableau 1:** Évolution du bilan phosphocalcique de notre patient

	4/12/15	14/12/15	18/02/16	07/03/16	20/05/16	09/08/16
Calcium mg/l (N= 85 – 102)	36	99	69	82	101	99
Phosphore mg/l (N= 30 – 45)	-	-	42	47	31	54
PTH pg/ml (N= 10 – 65)	-	-	684,5	463	327	113,7
25-OH Vit D pg/ml (N= 15,9 – 55,6)	-	-	15,1	18,7	31,4	33,3

## Discussion

Définie par Albright en 1942, la pseudohypoparathyroïdie d'Albright est une pathologie rare, associée à des troubles du bilan phosphocalcique liés à une résistance périphérique à la parathormone [1]. Il s'agit d'une affection héréditaire, transmise sur le mode autosomique dominant, dont la pénétrance et l'expression sont variables en fonction du sexe du parent transmetteur [1-3]. De ce fait, l'empreinte génomique parentale de la maladie est plus sévère lorsqu'elle est transmise par la mère des patients. Au cours de la pseudohypoparathyroïdie d'Albright, les études génétiques ont mis en évidence une mutation dans des cellules somatiques postzygotiques du gène guanine nucleotide binding protein, alpha-stimulating activity polypeptide 1 (GNAS1), située sur le bras long (q13.2) du chromosome 20 [2]. Cette anomalie moléculaire aboutit, in vitro, à des concentrations réduites de la protéineGSa au niveau des érythrocytes (de l'ordre de 50%) [4]. Chez les patients atteints de pseudohypoparathyroïdie d'Albright, la concentration sérique en calcium est faible et la phosphorémie est élevée (Tableau 2), comme dans notre observation. L'augmentation du taux de parathormone sérique reflète une résistance à l'action de cette hormone [1]. Les autres axes hormonaux peuvent également être touchés avec, principalement, une hypothyroïdie par résistance à la thyréostimuline hormone (TSH) et un hypogonadisme par résistance à l'hormone lutéostimulante (LH) et laFolliculo-stimuline hormone (FSH) [5]. La plupart des patients ayant une pseudohypoparathyroïdie d'Albright présentent un syndrome dysmorphique caractéristique, comportant, selon des degrés divers [6]; anomalies cervico-céphaliques: faciès arrondi (90%), cou court, racine du nez aplatie, hypoplasie dentaire; petite taille (80%), obésité (90%); anomalies osseuses: brachymétacarpie (70%), brachymétatarsie (40%); anomalies oculaires: hypertélorisme, microphtalmie, anisocorie, nystagmus, strabisme, diplopie; calcifications sous-cutanées; manifestations neurologiques: retard mental (75%), crises convulsives généralisées (60%) comme chez notre patient; autres anomalies squelettiques: raccourcissement du cubitus, cubitus valgus; déformation du radius, comme dans notre observation; déformation en coxa vara ou coxa valga; genu varum ou valgum; hyperostose crânienne frontale interne, amincissement de la voûte du crâne, craniosténose.

**Tableau 2:** Classification des pseudohypoparathyroïdies

Types de pseudo-hypoparathyroïdie	Ostéodystrophie d’Albright	Résistance hormonale multiple	Anomalies biologiques *	Test à la PTH		Défaut génétique
				AMPc urinaire	Phosphaturie	
**Ia**	Oui	Oui	Oui	Pas d’augmentation	Pas d’augmentation	
**Pseudopseudo-hypoparathyroïdie**	Oui	Inconstante	Non	Augmentation	Augmentation	Anomalie du gène GNAS-unité 1 codant pour la sous-unité α de la protéine G stimulatrice ; empreinte génomique
**Ib**	Non	Non	Oui	Pas d’augmentation	Pas d’augmentation	Anomalies d’un promoteur du gène du récepteur de la PTH/PTHrP?
**Ic**	Oui	Oui	Oui	Pas d’augmentation	Pas d’augmentation	sous-unité α de la protéine G stimulatrice normale; anomalie de l’adénylate cyclase
**II**	Inconstante	Non	Oui	Augmentation	Pas d’augmentation	Anomalie d’un second messager intracellulaire?

PTH: parathormone; PTHrP: parathyroid hormone-related peptide;

* : hypocalcémie, élévation de la PTH

La confirmation du diagnostic de l'ostéodystrophie héréditaire d'Albright se fait par le test à la parathormone [7] qui permet d'évaluer la réceptivité périphérique des tissus cibles à la PTH exogène et définir ainsi la résistance à la PTH en l'absence de réponse plasmatique et urinaire des marqueurs de réponse. II est réalisé par l'injection de 100 Ulm2 de PTH exogène (ParatharB), puis la mesure du taux d'AMPc plasmatique à TO, T5, Tl 0, T60 associée à une évaluation urinaire. La réponse plasmatique est considérée comme normale si le taux pic est supérieur à 8 fois le taux de base. Nous n'avons pas pu effectuer ce test chez notre patient. L'étude génétique permet de confirmer le diagnostic par la recherche d'une mutation dans le gène GNAS1 [8]. Aucun traitement spécifique n'a été décrit. Néanmoins, une supplémentation calcique est préconisée durant les épisodes d'hypocalcémie symptomatiques, en association avec la vitamine D, pour permettre une absorption intestinale du calcium et rétablir l'équilibre phosphocalcique. Les lésions cutanées peuvent bénéficier d'un traitement chirurgical et le traitement par L-Thyroxine permet de suppléer une dysfonction thyroïdienne en cas de résistance à la TSH [9]. Une surveillance, clinique est justifiée, notamment concernant le développement staturopondéral, en prévenant tout particulièrement le risque de surpoids ou d'obésité, ainsi que le développement psychomoteur, l'existence de signes orientant vers une hypocalcémie. Sur le plan biologique, une surveillance régulière du bilan phosphocalcique est recommandée, d'autant plus qu'un traitement substitutif est mis en place. La normalisation de la calcémie est l'objectif thérapeutique étant donné qu'il prévient l'hyperparathyroïdie secondaire.

## Conclusion

L'ostéodystrophie héréditaire d'Albright fait partie des pathologies rares et méconnues qui peuvent mettre énormément de temps à être découvertes et ne bénéficient pas de traitement spécifique. Cette observation soulève la nécessité d'explorer toute hypocalcémie profonde et/ou persistante chez le nouveau-né.

## Conflits d’intérêts

Les auteurs ne déclarent aucun conflit d'intérêts.
